# The Cognitive Side of M1

**DOI:** 10.3389/fnhum.2016.00298

**Published:** 2016-06-17

**Authors:** Barbara Tomasino, Michele Gremese

**Affiliations:** IRCCS “E. Medea”San Vito al Tagliamento, Italy

**Keywords:** primary motor cortex, M1, fMRI, cognitive processing, ALE meta-analysis

## Abstract

The primary motor cortex (M1) is traditionally implicated in voluntary movement control. In order to test the hypothesis that there is a functional topography of M1 activation in studies where it has been implicated in higher cognitive tasks we performed activation-likelihood-estimation (ALE) meta-analyses of functional neuroimaging experiments reporting M1 activation in relation to six cognitive functional categories for which there was a sufficient number of studies to include, namely motor imagery, working memory, mental rotation, social/emotion/empathy, language, and auditory processing. The six categories activated different sub-sectors of M1, either bilaterally or lateralized to one hemisphere. Notably, the activations found in the M1 of the left or right hemisphere detected in our study were unlikely due to button presses. In fact, all contrasts were selected in order to eliminate M1 activation due to activity related to the finger button press. In addition, we identified the M1 sub-region of Area 4a commonly activated by 4/6 categories, namely motor imagery and working memory, emotion/empathy, and language. Overall, our findings lend support to the idea that there is a functional topography of M1 activation in studies where it has been found activated in higher cognitive tasks and that the left Area 4a can be involved in a number of cognitive processes, likely as a product of implicit mental simulation processing.

## Introduction

The human primary motor cortex (M1) lies in the anterior bank of the precentral sulcus and its primary role is to control body parts movement. M1 also participates, for some aspects, in sensorimotor transformation rather than simply controlling the parameters of movement execution (Schieber, 2000). Indeed, studies using single-cell recording in monkeys (Ashe et al., 1993; Pellizzer, 1996; Carpenter et al., 1999; Wise and Murray, 2000) have lent support to the idea that M1 can be involved in higher motor functions, too. This also emerged from brain imaging studies (Grafton et al., 1995; Porro et al., 1996; Honda et al., 1998; Karni et al., 1998) and transcranial magnetic stimulation (TMS) studies in humans (Ganis et al., 2000; Tomasino et al., 2005, 2008). These studies suggested that M1 does not only play a role in stimulus-response compatibility, plasticity, motor sequence learning and memory as well as learning of sensorimotor associations but is engaged in motor imagery and spatial transformations. Neurophysiological studies showed that different neuronal population discharge patterns in M1 reflect several types of information such as spatial goals, hand motion direction (Georgopoulos et al., 1989; Georgopoulos and Pellizzer, 1995), muscular force and the global goal of the task, (e.g., Scott and Kalaska, 1997; Kakei et al., 1999). Some cells are sensitive to changes in limb posture, i.e., to the direction of movement depending on the actual position of an arm in space (Caminiti et al., 1990), while others receive sensory inputs (Scott, 1997). In addition, it has been shown that cortical stimulation of M1 in wide-awake monkeys evokes complex postures (Graziano et al., 2002). Studies specifically related to mental rotation showed that M1 plays a role in visuomotor transformations (Georgopoulos et al., 1989). In particular, recent fMRI studies have shown that M1 may be involved in tasks tapping empathy, and a direct correlation between sensorimotor activation and empathy for pain has been found (Lamm et al., 2007). In addition, M1 has been found activated during emotion processing, with participants silently reading emotional words, yet in a strategy-dependent manner (Papeo et al., 2012). Working memory tasks such as remembering sensory material or remembering finger movement sequences after a time delay (Kaas et al., 2007) have been found to activate M1, too, similarly to motor imagery and mental rotation tasks, (e.g., Porro et al., 1996; Kosslyn et al., 2001). Lastly, M1 activation has been reported during language processing of action-related words (Pulvermuller et al., 2001; Hauk et al., 2004; Pulvermuller, 2005) unexpectedly adding a new linguistic dimension to this area (de Lafuente and Romo, 2004). Furthermore, it is known that M1 can be divided into two separate and structurally different areas, namely Area 4a and Area 4p which differ in their cytoarchitecture and neurochemistry (Geyer et al., 1996). Cytoarchitectonic Area 4a (Geyer et al., 1996) is the rostral part of M1 and receives more extensive cortico-cortical projections from area six than the more posterior Area 4p which is more connected with somatosensory areas, see (e.g., Geyer et al., 1996). In relation to the functional aspects of these sub-areas, it has been suggested that the two areas differ functionally (Geyer et al., 1996). It has been shown that area 4p was more activated by movements guided by somatosensory information, whereas area 4a was more activated by externally triggered movements (Geyer et al., 1996), and that activation of area 4p was modulated by attention to action, while 4a was not (Binkofski et al., 2002). Authors (Binkofski et al., 2002) argued that Area 4a and Area 4p may belong to different motor channels allowing for parallel processing of motor information with different attention load. In particular, Area 4p might reflect increased attention to sensory feedback, whereas Area 4a might be responsible for maintaining the execution of a motor program, irrespective of the amount of attention paid to it. M1 is known to be involved in action planning and execution together with the premotor cortex, the supplementary motor area, the posterior parietal cortex and subcortical areas (Binkofski et al., 2002). Here, we use the term executive function to refer to processes involved in execution of motor events, and cognitive to refer to non-motor execution such as for example mental rotation or action words processing. In a further study it has been shown that imagery of a finger opposition sequence activated both 4a and 4p with a higher involvement of area 4p and authors argued that this activation reflected spatial encoding (Sharma et al., 2008).

In order to test the hypothesis that there is a functional topography of M1 activation (both in the left and the right hemisphere) in studies where it has been implicated in higher cognitive tasks, we performed quantitative activation-likelihood-estimation (ALE) meta-analyses (Eickhoff et al., 2012) of functional neuroimaging experiments in which M1 has been found activated by several tasks. In particular we addressed six categories, those for which we reached a sufficient number of studies to be included, namely: motor imagery, working memory, mental rotation, emotion/empathy, semantic and lexical decision, and auditory processing, in which M1 has been found activated, for a total of 126 imaging experiments, with 1818 subjects and 2030 activation foci.

## Materials and methods

We searched the PubMed database (http://www.ncbi.nlm.nih.gov/pubmed/), the Web of Knowledge database (http://www.webofknowledge.com) and the Sleuth on-line database (http://brainmap.org) for functional neuroimaging experiments that featured at least one cluster on the primary motor cortex. We identified paradigms pertaining to 6 functional categories (see Table 1). The categories included: (i)social/emotion/empathy (empathy of pain, social cognition, e.g., subjects viewed a human hand in pictures depicting painful or non-painful situations or they processed emotional words), (ii) working memory (memory, e.g., n-back task, remembering the position of a dot), (iii) motor imagery (mental simulation of movements e.g., mental imagery of walking or of finger tapping), (iv) mental rotation (mental transformations, handedness decisions, object rotation e.g., mental rotation of objects, hands and alphanumeric characters), (v) language processing (semantic representation, action word processing e.g., silently reading action and non-action words or verbs), and (vi) auditory imagery and perception^1^ (music imagery, perception, action sounds e.g., passive listening to speech, songs, tones; See Table 1). We used the following search terms. Each of the following keywords: somatosensory, precentral, postcentral; somatomotor, M1, S1, was, in turn, combined to each pair of words, as follows. For the emotion/empathy category we added: empathy and MRI, empathy and pain, mentalizing and MRI, social cognition and MRI, theory of mind and MRI; for the action word processing field, we added: lexical decision, action words; for the mental rotation field, we added: mental rotation; for the motor imagery field, we added: motor imagery; for the working memory field, we added for working memory; for the auditory field, we added: auditory

**Table 1:** **Studies details**.

**N°**	**Field of study**	**References**	**fMRI-PET**	**Tesla**	**Subjects**	Sex^*^	**Handedness^∧^**	**Coordinate**	**Software**	**Contrasts**	**Motor response required by the task**	**Hand L/R both**	**Motor response required by the control condition**	**Hand L/R both**
1	Motor imagery	Lacourse et al., 2005	fMRI	1.5	54	35f	R	MNI	SPM	Previously learned movements>rest	–		–	
										Imaging new movements>rest	–		–	
2	Motor imagery	Lorey et al., 2011	fMRI	1.5	23	12f	R	MNI	SPM	High and low imaginary during mental imagery>rest	v	L	v	L
3	Motor imagery	Malouin et al., 2003	fMRI	1.5	6	5f	ND	Talairach	ND	Imagery of walk>rest	–		–	
										Imaging to perform a step forward and one backward>rest	–		–	
										Walk with obstacles>rest	–		–	
4	Motor imagery	Szameitat et al., 2007	fMRI	3T	15	6f	R	Talairach	SPM	Upper extremities movements+whole body movements>rest	–		–	
5	Motor imagery	Ueno et al., 2010	fMRI	1.5T	15	3f	R	MNI	SPM	Motor imagery>rest	–		–	
6	Motor imagery	Gemignani et al., 2004	fMRI	1.5T	6	6f	ND	Talairach	ND	(Movement imagery+movement execution)> control	v	R	v	R
7	Motor imagery	Tomasino et al., 2007	fMRI	1.5T	15	7f	R	MNI	SPM	(Imagery of motor - non motor verbs)> control	v	Counter–balanced	v	Counter–balanced
8	Motor imagery	Sharma et al., 2008	fMRI	3T	14	8f	R	MNI	SPM	Mental imagery>rest	–		–	
9	Motor imagery	Boecker et al., 2002	PET		6	3m	R	MNI	Sun Spark Station	Mental imagery>rest	–		–	
10	Motor imagery	Guillot et al., 2009	fMRI	3T	50	26f	R	MNI	SPM	Mental imagery-control	–		–	
										Kinestetic mental imagery>rest	–		–	
11	Motor imagery	Grezes and Decety, 2002	PET		10	m	R	Talairach	SPM	Motor imagery-control	v	Both	v	
12	Motor imagery	Stephan et al., 1995	PET	1T	6	m	R	Talairach	SPM	Imagined movement- control	–		–	
13	Motor imagery	Ehrsson et al., 2003	fMRI	1.5T	6	1f	R	MNI	SPM	Finger movement imagination> control1	–		–	
										Finger movement imagination> control2	–		–	
										Toe movement imagination> control2	–		–	
										Toe movement imagination> control1	–		–	
										Tongue movement imagination> control1	–		–	
										Tongue movement imagination> control2	–		–	
14	Working memory	Alain et al., 2008	fMRI	3T	16	8f	R	Talairach	AFNI	Location recognition> control	v	R	v	R
					16					Transient location recognition> control	v	R	v	R
					16					Transient category recognition> control	v	R	v	R
15	Working memory	Ulloa et al., 2008	fMRI	1.5T	14	1f	ND	MNI	Brain Voyager	Delay2>control	v	Both	v	Both
					14					Delay3>control	v	Both	v	Both
16	Working memory	Pau et al., 2013	fMRI	3T	14	Piano playes: 6f; musically naive:6f	ND	MNI	SPM	Piano player> control	–		–	
					14					Piano player> control	v	Both	v	Both
17	Working memory	Babiloni et al., 2004	fMRI	1.5T	15	ND	R	Talairach	Brain Voyager	Delay period>fixation cross	–		–	
18	Working memory	Binder and Urbanik, 2006	fMRI	1.5T	12	5f	R	Talairach	SPM	Two-back verbal condition >control	v	LR feet	v	LR feet
	Working memory				12					two-back non-verbal condition>control	v	LR feet	v	LR feet
19	Working memory	Simon et al., 2002	fMRI	1.5T	10	4f	R	Talairach	SPM	Delay task>control	v	R	v	R
20	Working memory	Coombes et al., 2011	fMRI	3T	15	7f	R	Both, MNI e Tailarach	AFNI	0.4 Hz High gain> control	v	R	v	R
					15					6.4 Hz High gain> control	v	R	v	R
21	Working memory	Postle, 2006	fMRI	3T	13	ND	R	MNI	ND	Delaly>rest	–		–	
22	Working memory	Jahanshahi et al., 2006	PET		8	m	R	MNI	SPM	Long> control	v	R	v	R
23	Working memory	Ricciardi et al., 2006	fMRI	1.5T	6	m	R	Talairach	AFNI	Tactile maintainance>rest	–		–	
					6					Visual mainteinance>rest	–		–	
24	Working memory	Cairo et al., 2004	fMRI	1.5T	18	10f	ND	Talairach	SPM	Retrieval>rest	–		–	
25	Working memory	Honey et al., 2000	fMRI	1.5T	20	m	R	Talairach	Personal	N-back>rest	v	R	v	R
26	Working memory	Rama et al., 2001	fMRI	1.5T	8	f	R	Talairach	MedX	Experiment 1: Two-Back vs. control.	v	R	v	R
					8					Experiment 2: One-Back vs. control	v	R	v	R
27	Working memory	Dade et al., 2001	PET		12	6f	R	MNI	ND	two-back faces>control	v	N.R.	v	N.R.
					12					Two-back odor> control	v	N.R.	v	N.R.
28	Working memory	Kim et al., 2002	PET		14	7f	R	Talairach	SPM	n-back words>control	v	R	v	R
29	Working memory	Casey et al., 1998	fMRI	1.5T	5	ND	R	Talairach	AFNI	Memory> control1	v	R	v	R
					6					Memory> control2	v	R	v	R
					8					Memory> control3	v	R	v	R
30	Working memory	Jonides et al., 1997	PET		18	f	ND	Talairach	ND	Two-back>control	v	N.R.	v	N.R.
31	Working memory	Rypma et al., 2001	fMRI	1.5T	6	4f	ND	talairach	Sun Spark Station	N-back>control	v	R	v	R
32	Mental rotation	Kosslyn et al., 1998	PET		20	m	R	Talairach	SPM	Hand rotation> control	v	R Lfeet	v	R Lfeet
33	Mental rotation	Jordan et al., 2002	fMRI	1.5T	24	14f	R	MNI	SPM	Male rotation of letter-3D-abstract figures> control	v	R	v	R
34	Mental rotation	Suchan et al., 2006	fMRI	1.5T	11	5f	R	Talairach	SPM	Mental rotation simultaneous matrix rotation> control	v	N.R.	v	N.R.
										Successive matrix rotation> control	v	N.R.	v	N.R.
35	Mental rotation	de Lange et al., 2006	fMRI	3T	17	1f	R	Talairach	SPM	Right hand mental rotation> control	v	R L feet	v	R L feet
36	Mental rotation	Papeo et al., 2012	fMRI	3T	18	f	R	MNI	SPM	Motor imagery MR> control	v	R L feet	v	R L feet
37	Mental rotation	Vingerhoets et al., 2001	PET		10	5f	R	MNI	SPM	Alphanumeric rotation> control	v	both	v	both
38	Mental rotation	Wraga et al., 2010	fMRI	3T	18	10F	R	Talairach	SPM	MR of cubes> control	v	both	v	both
	Mental rotation									Perspective change - control	v	both	v	both
39	Mental rotation	Mourao-Miranda et al., 2009	fMRI	1.5T	11	11F	R	Talairach	SPM	MR > fixtion	v	N.R.	–	N.R.
40	Social/Emotion/Empathy/Self	Hooker et al., 2010	fMRI	4T	15	7f	ND	MRI	SPM	Social change> control	–		–	
41	Social/Emotion/Empathy/Self	Guo et al., 2012	fMRI	3T	16	11f	R	MNI	SPM	High pain evaluation> control	–		–	
42	Social/Emotion/Empathy/Self	Lamm et al., 2007	fMRI	3T	18	9f	ND	MNI	SPM	Empathy for intensity> control	v	R	v	R
43	Social/Emotion/Empathy/Self	Lutz et al., 2009	fMRI	3T	23	4f	R	Talairach	AFNI	Compassion image> control	–		–	
44	Social/Emotion/Empathy/Self	Brunet et al., 2000	PET		8	m	R	Talairach	SPM	Understand the man future intention> control	v	R	v	R
45	Social/Emotion/Empathy/Self	Vogeley et al., 2001	fMRI	1.5T	8	m	R	Talairach	SPM	Personal perspective>rest	–		–	
46	Social/Emotion/Empathy/Self	Moriguchi et al., 2007	fMRI	1.5T	14	ND	R	MNI	SPM	Painful images> control	v	R	v	R
47	Social/Emotion/Empathy/Self	Walter et al., 2004	fMRI	1.5T	13	7f	R	Talairach	SPM	Understand the intention> control	v	N.R.	v	N.R.
48	Social/Emotion/Empathy/Self	Rilling et al., 2004	fMRI	3T	19	11f	ND	Talairach	Brain Voyager	Both prigioners collaborate> control	v	N.R.	v	N.R.
49	Social/Emotion/Empathy/Self	Moseley et al., 2012	fMRI	3T	18	ND	R	MNI	SPM	Emotion word> control	–		–	
										All abstract emotion word> control	–		–	
50	Social/Emotion/Empathy/Self	Shafer and Dolcos, 2012	fMRI	1.5T	16	9f	R	Talairach	SPM	Emotional pictures short time presentation> control	v	N.R.	v	N.R.
51	Social/Emotion/Empathy/Self	Luo et al., 2006	fMRI		20	11f	ND	Talairach	AFNI	Legal image> control	v	both	v	both
52	Social/Emotion/Empathy/Self	(Kensinger and Schacter, 2005)	fMRI	1.5T	16	8f	R	Talairach	SPM	Accurate retrieval of emotional word and picture items(matched)> control	v	N.R.	v	N.R.
53	Social/Emotion/Empathy/Self	Lawrence et al., 2006	fMRI	1.5T	12	6f	R	Talairach	In house software	Perceiving the emotional state of the image> control	v	N.R.	v	N.R.
54	Social/Emotion/Empathy/Self	Domes et al., 2010	fMRI	1.5T	33	18f	R	MNI	SPM	Male(increase>maintain)> control	–		–	
55	Social/Emotion/Empathy/Self	Ochsner et al., 2005	fMRI	exp1:4T; exp2:3T	16	Exp1:9f Exp2: 9f	ND	MNI	SPM	Judge an adjective on itself> control	v	Both	v	Both
56	Social/Emotion/Empathy/Self	Izuma et al., 2008	fMRI	3T	10	1f	R	MNI	SPM	Other> control	v	R	v	R
57	Social/Emotion/Empathy/Self	Gu et al., 2013	fMRI	3T	18	9f	R	MNI	SPM	Pain evaluation with low retribution> control	v	N.R.	v	N.R.
58	Language	Tomasino et al., 2010	fMRI	3T	19	9f	R	MNI	SPM	Imperative with DO> control	v	L R feet	v	L R feet
59	Language	Moore-Parks et al., 2010	fMRI	3T	16	7f	R	Talairach	AFNI	Lexical decision>control	v	L	v	L
60	Language	Mellet et al., 1998	PET		8	m	R	Talairach	SPM	Concrete word definition> control	–		–	
61	Language	Carota et al., 2012	fMRI	3T	18	ND	R	MNI	SPM	Tool words> control	–		–	
										Food words> control	–		–	
62	Language	Peran et al., 2010	fMRI	3T	12	7f	R	MNI	SPM	Mentally simulate the content> control	–		–	
63	Language	Gitelman et al., 2005	fMRI	1.5T	15	7f	R	MNI	SPM	semantic>control	v	R	v	R
64	Language	Kemmerer et al., 2008	fMRI	1.5T	16	8f	R	MNI	SPM	Running verbs> control	v	L	v	L
										Hitting verbs> control	v	L	v	L
65	Language	Raposo et al., 2009	fMRI	3T	22	ND	R	MNI	SPM	Listen to action words> control	–		–	
										Listen to action arm word> control	–		–	
66	Language	Papeo et al., 2012	fMRI	3T	18	f	R	MNI	SPM	Silent reading of verbs> control	–		–	
67	Language	Postle et al., 2008	fMRI	4T	18	13f	R	MNI	SPM	Mouth words> control	–		–	
68	Language	de Diego Balaguer et al., 2006	fMRI	1.5T	12	8f	R	MNI	SPM	Covert regular inflection> control	–		–	
										Covert irregular inflection> control	–		–	
										Covert nonce inflection> control	–		–	
69	Language	Fliessbach et al., 2006	fMRI	1.5T	21	12f	R	Talairach	SPM	Recognition, old/New hit> control	v	N.R.	v	N.R.
70	Language	Assaf et al., 2006	fMRI	3T	18	7f	R	Talairach	SPM	Correct recall> control	v	R	v	R
										Correct non associated> control	v	R	v	R
71	Language	Haller et al., 2005	fMRI	1.5T	15	m	R	Talairach	Brain Voyager	Sentence generation> control	–		–	
72	Language	Addis and McAndrews, 2006	fMRI	1.5T	12	7f	R	MNI	SPM	Relational load (two-link> control	v	R	v	R
73	Language	Specht and Reul, 2003	fMRI	1.5T	12	2f	R	MNI	SPM	Word>rest	–		–	
74	Language	Berlingeri et al., 2008	fMRI	1.5T	12	6f	R	MNI	SPM	Verbs> control	–		–	
										Verbs> control)	–		–	
75	Language	Marangolo et al., 2006	fMRI	1.5T	10	5f	R	MNI	SPM	Verb inflection > control	–		–	
										Adjective inflection> control	–		–	
76	Language	Stringaris et al., 2007	fMRI	1.5T	11	m	R	Talairach	In house software	Literal> control	v	R	v	R
										Literal> control	v	R	v	R
										Non metaphoric> control	v	R	v	R
77	Language	Uchiyama et al., 2006	fMRI	3T	20	10f	R	MNI	SPM	Situation> control	v	R	v	R
78	Language	Schmidt and Seger, 2009	fMRI	3T	10	5f	R	Talairach	Brain Voyager	Easy-familiar+easy-unfamiliar+difficult-unfamiliar methaphors> control	v	N.R.	v	N.R.
79	Language	Cansino et al., 2002		2T	17	15f	R	Talairach	SPM	Correct source memory during classification of natural vs. artificial items (encoding phase) > control	v	N.R.	v	N.R.
80	Auditory	Shergill et al., 2001	fMRI	1.5T	8	m	ND	Talairach	ND	First person auditory verbal imagery> control	–		–	
										Second person auditory verbal imagery> control	–		–	
										Third person auditory verbal imagery> control	–		–	
										Combined imagining speech> control	–		–	
81	Auditory	Jardri et al., 2007	fMRI	1.5T	12	m	R	Talairach	Brain Voyager	Unfamiliar voice> control	–		–	
										Unfamiliar voice> control	–		–	
										Unfamiliar voice> control	–		–	
82	Auditory	Pastor et al., 2002	PET		9	4f	R	Talairach	SPM	Hearing tones at different Hz>rest	–		–	
83	Auditory	Brown and Martinez, 2007	fMRI	2T	11	6f	R	Talairach	In house software	Melodies comparation>control	v	R	v	R
										Harmony comparation>control	v	R	v	R
84	Auditory	Callan et al., 2006	fMRI	3T	16	5f	R	Talairach	SPM	Listen singing > control	–		–	
										Covert speech> control	–		–	
85	Auditory	Poeppel et al., 2004	PET		10	5m	R	Talairach	SPM	Categorical perception>control	v	Both	V	Both
										Discrimination of frequences> control	v	Both	V	Both

*Sub-sectors of M1 showing significant relative increases in BOLD response associated with each category. In the whole brain analysis part of the table we reported the cluster of activations found in the whole brain analysis clearly indicating an activation of Area 4. As these activations often spread to the sensory cortex or to the premotor cortex, in the ROI analysis we report the cluster of activations found after having performed a ROI analysis on the whole brain analysis results by using a mask of M1*.

* F, females; m, males;

∧ *R, right-handed, L, left-handed; N.R. not reported*.

Moreover, the literature cited in the filtered papers and review articles was also assessed to identify additional neuroimaging studies in which the M1 was found to be activated by different cognitive/emotive tasks.

The region of interest (ROI) corresponded to M1 and was defined by using the SPM Anatomy toolbox (Eickhoff et al., 2005) to derive the anatomically-constrained ROIs of M1 (Geyer et al., 1996). The anatomical masks were created by using the “create anatomical ROIs” function of the Anatomy toolbox and by selecting areas 4a and 4p of the left and the right hemisphere (see Supplementary Figure 1).

Our meta-analysis included studies with PET or fMRI experiments on healthy subjects and excluded pharmacological trials or studies involving clinical populations. The reason for including PET studies was that, although they might have very different temporal and spatial sensitivity and resolution than fMRI, their inclusion increased the size of each category included in the analysis. Altogether, PET studies in the motor imagery category were 3/22 (13.63%), in memory were 4/29 (13.79%), in mental rotation were 2/10 (20%), in social /emotion/empathy/self were 1/19 (5.2%), in language were 1/32 (3.1%), and in auditory were 2/14 (14.2%).

To date, when possible, we checked whether there was any concordance between fMRI and PET studies using similar tasks or paradigms. For instance, in the motor imagery category both the study by Lacourse et al. (2005) and Boecker et al. (2002) used imagery of finger sequences and find activation in the frontal (e.g., superior frontal gyrus, precentral/premotor cortex), parietal (postcentral/sensorimotor cortex, inferior, and superior parietal lobe), and cererbellum and subcortical (putamen).

All participants were right handed except for 1 study involving a left hander and some studies in which this information was not reported. All single-subject reports were excluded.

We excluded a total of 331 studies that didn't report any cluster within the M1, and 45 that although argued to have found activation in M1, did not report the corresponding coordinates, 31 patients studies and 5 pharmacological studies, and one study including younger subjects. In addition, only studies which reported the coordinates in a standard reference space (Talairach/Tournoux, MNI) were considered. Differences in coordinate space (MNI vs. Talairach space) were accounted for by transforming coordinates reported in Talairach space into MNI coordinates by a linear transformation (Lancaster et al., 2007). We only (when necessary) converted from Talaiarch to MNI coordinates (and not vice versa).

For each category, we only included studies which eliminated activations solely due to motor responses, for example, a button press. If a task required a button press, we made sure that the response was required in both the task and in the control condition (see Table 1 the columns labeled “Motor response required by the task” and “Motor response required by the controls condition”). Many of the included studies did not require button press. All the other studies required a button press both in the task and in the control condition. For example, in one of the studies (Lorey et al., 2011), it is reported that “During MI, participants […] when imagery was over and the button had been pressed. In the rest condition, participants also pressed a button at the beginning and at the end of the rest trial with their left hand.” In (Moore-Parks et al., 2010)'s study, subjects were asked to listen to a phrase and push two different buttons according to whether the phrase made sense or not. In the control task, participants listened to reverse phrases and pushed a button. This approach enabled us to exclude all brain activations in M1 due to button press. In addition, we included a further column in Table 1 indicating the hand used for but presses. In particular, we checked whether the use of the right (dominant) hand could have introduced some bias in the lateralization of activations in the final ALE maps (see results).

Based on these criteria, we included a total of 126 experiments from 85 papers for a total of 1818 subjects as and 2030 activations. A table describing all the included studies can be found in Table 1.

Closely following Stoodley and Schmahmann's approach used in an ALE meta-analysis of cerebellum-related functions (Stoodley and Schmahmann, 2009) we first performed six separate ALE-analyses. Each of the above categories was separately analyzed in order to obtain the activation clusters related to each experimental paradigm. Then, to investigate whether activation in a subpart of M1 was shared by all the categories, i.e., functional integration (Kurth et al., 2010), we used the results from the conventional ALE and a conjunction approach (see below).

### Statistical procedure

The meta-analysis was performed by using the revised version (Eickhoff et al., 2012) of the activation likelihood estimation (ALE) approach for coordinate-based meta-analysis of neuroimaging results.

To account for the uncertainty that is technically inherent to the actual location of the peaks, the method allows for modeling each coordinate not as a single point, but by a three-dimensional (3D) Gaussian function with 12 mm full-width half-maximum (FWHM) (Eickhoff et al., 2012). Accordingly, the localization probability distributions describe the probability that a given focus actually lay within a particular voxel. Statistical significance was determined using a permutation test of randomly generated foci, using the same FWHM and number of foci. The voxel-wise comparison was tested against the null hypothesis of uniformly distributed peaks, giving a set of ALE-values necessary for thresholding the probability map. ALE probability maps were then thresholded at *p* < 0.05 (cluster level corrected for multiple comparisons; Eickhoff et al., 2012) and a minimum cluster size of 200 mm^3^ was set.

We first performed five separate ALE-analyses. For each category, the reported coordinates for functional activations were analyzed for topographic convergence using the ALE method and the results were mapped on M1. The “Social-Emotion-Empathy” analysis included 400 activation foci (312 subjects and 19 experiments), the “Working Memory” analysis included 663 activation foci (351 subjects and 29 experiments), the “Motor Imagery” analysis included 258 activation foci (372 subjects and 22 experiments), the “Mental Rotation” analysis included 60 activation foci (158 subjects and 10 experiments), the “Language processing” analysis included 387 activation foci (474 subjects and 32 experiments) and the “Auditory” analysis included 262 activation foci (151 subjects and 14 experiments).

An anatomical mask of M1 in MNI space was created by using the SPM Anatomy toolbox (Eickhoff et al., 2005) to derive the anatomically-constrained ROI of the primary motor cortex (Geyer et al., 1996). We used the M1 ROI (see above and Supplementary Figure 1) to mask the resulting activation from the different ALE meta-analyses. Thereafter, we considered only the voxels of the ROI that were located within the cytoarchitectonically defined maximum probability maps (MPMs) of M1 (Brodmann Area 4). Activations within this mask were displayed on a rendered template brain (Colin27_T1_seg_MNI) provided by Gingerale (http://www.brainmap.org/ale/). Activations were also assigned histologically using the SPM Anatomy Toolbox (Eickhoff et al., 2005). The latter approach was important in order to eliminate activation of, e.g., the premotor cortex spilling over into the M1 mask.

Secondly, we tested whether the M1 cluster was conjointly activated by all the categories. The resulting shared area was identified by calculating the conjunction between the ALE files of each category. We used the FSL (http://fsl.fmrib.ox.ac.uk) to calculate which voxels were commonly activated by all the six categories to show crude overlap (it does not allow to make statistical claims). The selected mathematical operation enabled us to transform each activation output file in a binary matrix and then perform a mathematical matrix sum. If the sum of a matrix cell was 6, the corresponding voxel was active in all the six categories. If the sum was 5, it was active in five out of six categories, and so on.

## Results

**Figure 1:**
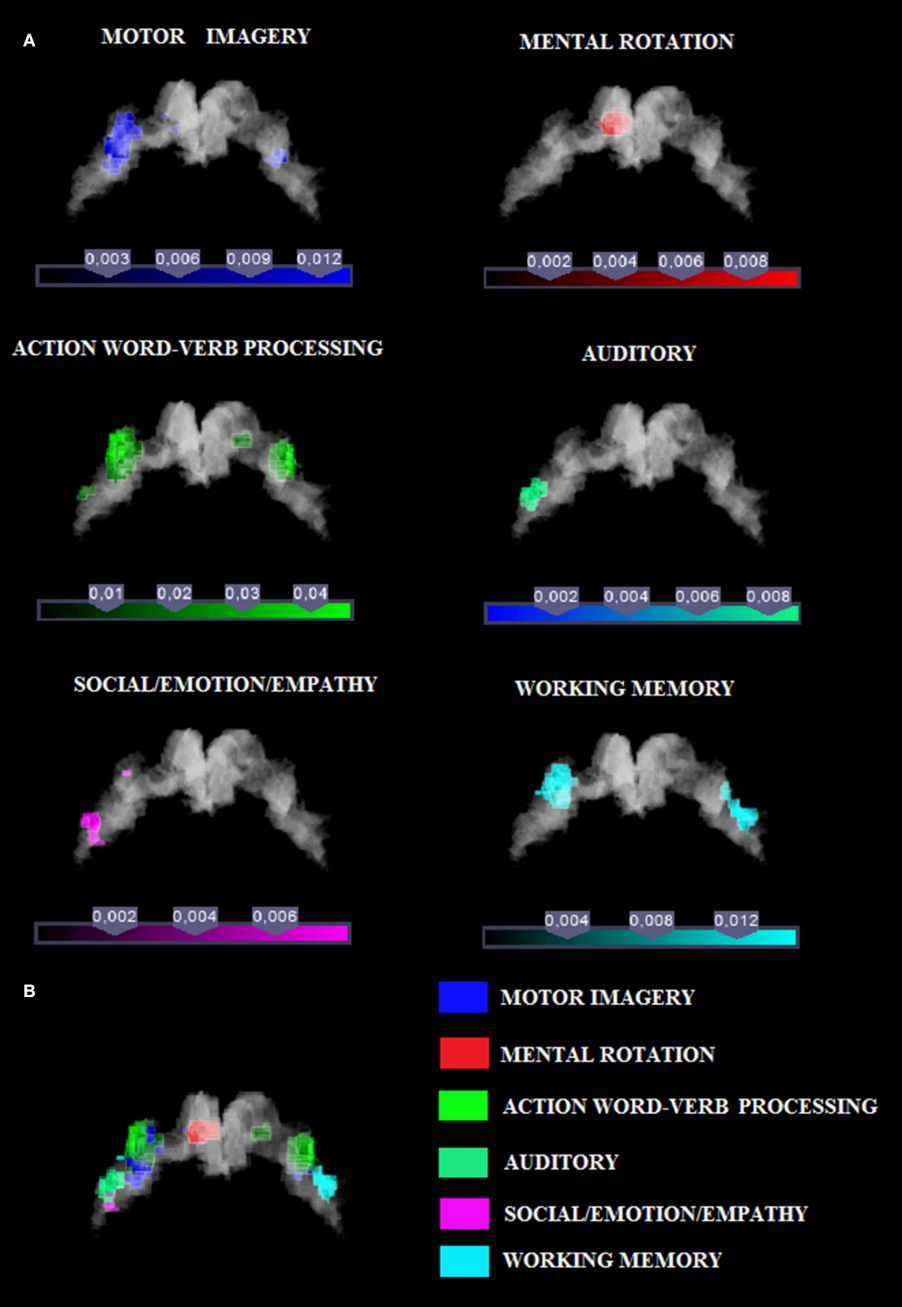
**(A)** Relative increases in neural activity associated with each category are displayed separately on a coronal slice of the anatomical mask of M1 created by using the SPM Anatomy toolbox (Eickhoff et al., 2005). In yellow it is shown the mask of Area 4p. The remaining part of M1 is obviously area 4a. **(B)** The overlay of all the categories evidencing the functional topography in M1. Color bar shows ALE value.

In this study we investigated the functional organization of the human motor cortex (Brodmann Areas 4a–4p; Geyer et al., 1996) by analyzing coordinates from functional neuroimaging experiments (See Table 1 for a list of 85 studies and a total of 126 experiments) that featured at least one cluster on the primary motor cortex. We identified six cognitive functional categories, namely (i) social/emotion/empathy, (ii) working memory, (iii) motor imagery, (iv) mental rotation, (v) language processing, and (vi) auditory imagery and perception. In addition, we identified the M1 sub-region commonly activated by four out of six categories. The results of all our meta-analyses are shown in Table 2. In all our analyses (see Figure 1; Supplementary Figure 2 showing results from the whole brain analysis), we found that activations for each of the six categories were rather confined to a subpart of M1, suggesting the presence of a common area in M1 shared by all categories. In addition, we found that some of the six categories activated M1 bilaterally and some tasks triggered a left- or right-lateralized activation (see below).

**Table 2:** **List of significant ALE foci**.

**Whole brain analysis**						**ROI analysis**					
**Region**	**Side**	**MNI**			**Cluster size**	**Region**	**Side**	**MNI**			**Cluster size**
		***x***	***y***	***z***	**Voxel**			***x***	***y***	***z***	**Voxel**
**SOCIAL/EMOTION /EMPATHY**											
Area 4a	L	−46	−4	34	121	Area 4a 7%	L	−50	−10	42	48
Area 4a	L	−36	−22	64	60	Area 4a 28%	L	−36	−24	62	4
**ACTION WORD/VERB PROCESSING**											
Area 4p 20%; Area 4a 40%	L	−38	−24	58	607	Area 4p 11%; Area 4a 58%	L	−38	−24	58	243
Area 4a	R	38	−22	58	454						
Area 4p 30%; Area 4a 20%	R	18	−28	64	53	Area 4a 17% 4p 20%	R	38	−22	58	146
Area 4a	L	−54	−8	42	54	Area 4p 20%; Area 4a 8%	R	20	−28	64	33
**MENTAL ROTATION**											
Area 4a	L	−10	−28	64	120	Area 4a 49%	L	−10	−28	64	102
**WORKING MEMORY**											
Area 4a	R	50	−14	44	276	Area 4a 23%	L	−34	−24	60	173
						Area 4a 31%	R	50	−14	44	84
**MOTOR IMAGERY**											
Area 4a	L	−8	−20	70	661	Area 4a 12%	L	−10	−24	62	6
Area 4p 40%, Area 4a 40%	L	−36	−20	52	467	Area 4p 32%, Area 4a 10%	L	−36	−20	52	189
Area 4a	L	−32	−24	66	467	Area 4a 33%	R	14	−26	66	2
Area 4a 30%, Area 4p 20%	L	−36	−12	44	467	Area 4a 21%	R	36	−16	50	28
**AUDITORY**											
Area 4a	L	−48	−14	44	256	Area 4a 29%	L	−48	−12	44	62
Area 4a	L	−44	−4	50	256						

*For each region of activation, the coordinates in MNI space are provided in reference to the maximally activated voxel within an area of activation, as indicated by the highest Z-value (p < 0.05, corrected for multiple comparisons at the cluster level, height threshold p < 0.001, uncorrected). L/R = left/right*.

### Social/emotion/empathy associated with anterior region of M1

Activation related to tasks involving social, emotion processing, and empathic tasks converged on a cluster in the left Area 4a (see Figure 1 and Supplementary Figure 3 for sagittal slices and Table 2). The ROI analysis performed to restrain activation on the M1 mask further confirmed that those coordinates belonged to the anatomically-constrained ROIs of M1 (Geyer et al., 1996) (see right side of the Table 2). The empathy whole brain network found in this meta-analysis (See Supplementary Table S2) included activations in the left amygdala, left thalamus, right parahippocampal gyrus, left SMA, left superior medial gyrus. These areas are in line with the commonly activated network for emotion-empathy tasks, (e.g., Lamm et al., 2007).

### Working memory associated with anterior region of M1

Tasks involving working memory like the n-back task activated three main clusters in the left and the right 4a (see Figure 1 and Supplementary Figure 3 for sagittal slices and Table 2). The ROI analysis performed to restrain activation on the M1 mask further confirmed that those coordinates belonged to the anatomically-constrained ROIs of M1 (Geyer et al., 1996) (see right side of the Table 2). Working memory activations (See Supplementary Table S3) matched the commonly reported working memory brain network, (i.e., Kaas et al., 2007) thus they showed activations in the left postcentral gyrus, left SMA, left inferior and superior parietal lobule, right inferior parietal lobule, right angular gyrus, left insula, right middle frontal gyrus, right inferior frontal gyrus, right middle frontal gyrus, left middle frontal gyrus.

### Motor imagery associated with anterior and posterior region of M1

Activation related to motor imagery tasks was found in clusters assigned to area 4a and to 4p bilaterally (see Figure 1 and Supplementary Figure 3 for sagittal slices and Table 2). The ROI analysis performed to restrain activation on the M1 mask further confirmed that those coordinates belonged to the anatomically-constrained ROIs of M1 (Geyer et al., 1996; see right side of the Table 2). In addition, the whole brain motor imagery activation network (See Supplementary Table S4) found in this meta-analysis included activations in the right precentral gyrus, the left SMA, the left putamen, the left inferior parietal lobule, the left cerebellum, and the right insula. These areas are in line with the commonly activated network for the motor imagery task, (e.g., Porro et al., 1996).

### Mental rotation associated with anterior region of M1

We found two clusters of activation in the left area 4a (see Figure 1 and Supplementary Figure 3 for sagittal slices and Table 2). The ROI analysis performed to restrain activation on the M1 mask further confirmed that those coordinates belonged to the anatomically-constrained ROIs of M1 (Geyer et al., 1996; see right side of the Table 2). In addition, our meta-analysis on the whole brain revealed (See Supplementary Table S5) that the mental rotation network included the left postcentral gyrus, the left inferior parietal lobule, the right superior parietal lobule, the right postcentral gyrus, the left inferior occipital gyrus, and the left paracentral lobule. These activations are in line with the commonly accepted mental rotation network, (e.g., Kosslyn et al., 2001).

### Language processing associated with anterior and posterior region of M1

Activation by linguistic processing of action-related words and verbs was found clusters in areas 4a–4p bilaterally (see Figure 1 and Supplementary Figure 3 for sagittal slices and Table 2). The ROI analysis performed to restrain activation on the M1 mask further confirmed that those coordinates belonged to the anatomically-constrained ROIs of M1 (Geyer et al., 1996; see right side of the Table 2). In addition, our meta-analysis (See Supplementary Table S6) found activation in the left postcentral gyrus, the left inferior parietal lobule, the right superior parietal lobule, the right postcentral gyrus, the left inferior occipital gyrus, and the left paracentral lobule. All of these activations are in line with the commonly accepted verb processing related network, (e.g., Crepaldi et al., 2013).

### Auditory associated with anterior region of M1

We found activations in the left area 4a (see Figure 1 and Supplementary Figure 3 for sagittal slices and Table 2). The ROI analysis performed to restrain activation on the M1 mask further confirmed that those coordinates belonged to the anatomically-constrained ROIs of M1 (Geyer et al., 1996) (see right side of the Table 2). In addition, our meta-analysis on the whole brain (See Supplementary Table S7) revealed an auditory–related network which included the right SMA, the left superior temporal gyrus, the left insular lobe, the right superior temporal gyrus, the right inferior frontal gyrus, the left supramarginal gyrus, the left middle temporal gyrus, the left inferior frontal gyrus, the left caudate nucleus, and the left Heschls gyrus (Brown and Martinez, 2007).

### M1 lateralization and laterality of button presses

In the case in which we found a left lateralized effect (Social-Emotion-Empathy, Mental rotation and Auditory), there could be some bias driven by the hand used for respond, thus we checked the frequency of use of the right (dominant) hand in the different categories.

In particular, for the Social-Emotion-Empathy category, 33% of the studies involving a keypress required a right hand response, 16% left and right hand (50% were not reporting the information related to the side of key press). For the Mental Rotation category, 10% of the studies involving a keypress required a right hand response, 60% both left and right hand (30% of the studies were not reporting the information). For the Auditory category, 50% of the studies involving a keypress required a right hand response, 50% both left and right hand.

Thus, it is unlike that the lateralization effects are driven by the hand used to respond. This is evident also analyzing the other categories for which a bilateral activation of M1 was found despite a preponderance of right hand key presses. For the Working Memory category, 65% of the studies involving a keypress required a right hand response, 21% both left and right hand (13% of the studies were not reporting the information). For Motor imagery 25% of the studies involving a keypress required a right hand response, 25% a left hand response the 50% both left and right hand. For Language processing 53% of the studies involving a keypress required a right hand response, 20% a left hand response, 6% required the use of both left and right hand (20% of the studies were not reporting the information; See Supplementary Table S1).

### Shared M1 activation: Conjunction analysis

**Figure 2:**
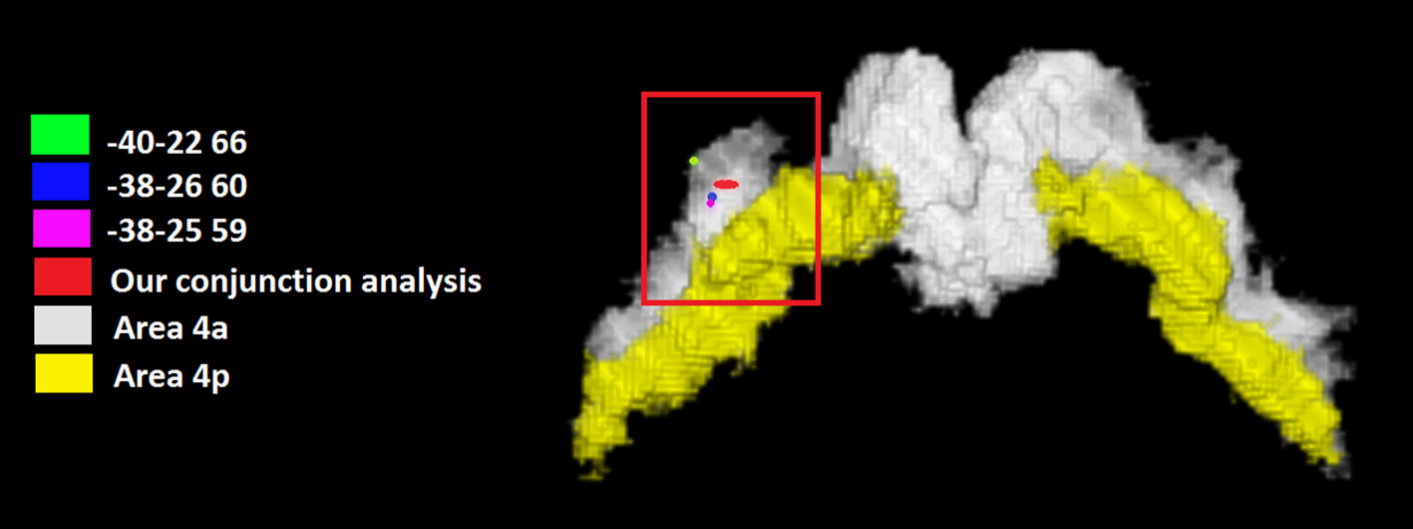
**The coordinates (in red) of the conjunction analysis showing the M1 sector conjointly activated by all the tested categories**. This area was situated in area 4 and was attributable to the hand area. We reported the local maxima found in some previous studies of our group in which a hand motor localizer task was used. In particular, we added three ROIs drawn on the mean MNI coordinates centered on the *x, y*, and *z* coordinates derived from the hand movement localizer task, averaged across participants, with the following coordinates: *x* = −38, *y* = −26, *z* = 60 (Papeo et al., 2012); *x* = −38, *y* = −25, *z* = 59 (Tomasino et al., 2010) and *x* = −40, *y* = −22, *z* = 66 (Tomasino et al., 2014). In yellow it is shown the mask of Area 4p.

We tested the possibility that one or more M1 regions were conjointly activated by all the tested categories. The conjunction analysis using the FSL program (http://fsl.fmrib.ox.ac.uk/fsldownloads/) showed a single cluster activation centered at the MNI coordinates *x, y, z*: −35, −25, +62, assigned to the left precentral gyrus, with a 84.4% probability for area 4a was found by setting the threshold of minimum cluster activation common to 4/6 of the considered categories, namely motor imagery and working memory, social/emotion/empathy, and language (see Figure 2). This area was situated in area 4 and was attributable to the hand area (Geyer et al., 1996). We reported in Figure 2 the local maxima found in some previous studies of our group in which a hand motor localizer task was used. In particular, we added three ROIs drawn on the mean MNI coordinates centered on the *x, y*, and *z* coordinates derived from the hand movement localizer task, averaged across participants, with the following coordinates: *x* = −38, *y* = −26, *z* = 60 (Papeo et al., 2012); *x* = −38, *y* = −25, *z* = 59 (Tomasino et al., 2010; and *x* = −40, *y* = −22, *z* = 66 (Tomasino et al., 2014). The coordinates of the conjunction analysis are located very close to these three local maxima, differing only 3, 1, and 2 mm from the coordinates of Papeo et al. (2012), only 3, 0, and 3 mm from the coordinates of Tomasino et al., 2010 and only 5, 3, and 6 mm from the coordinates of Tomasino et al. (2014).

## Discussion

M1 is traditionally implicated in voluntary movement control. However, the view that M1 can also be involved in higher motor functions has been purported by studies using different techniques such as single cell recording in monkeys (Ashe et al., 1993; Pellizzer, 1996; Carpenter et al., 1999; Wise and Murray, 2000), brain imaging (Grafton et al., 1995; Porro et al., 1996; Honda et al., 1998; Karni et al., 1998), and transcranial magnetic stimulation (TMS) techniques in humans (Ganis et al., 2000; Tomasino et al., 2005, 2008). These studies suggest that M1 is engaged in motor imagery and spatial transformations in addition to stimulus–response compatibility, plasticity, motor sequence learning and memory, learning of sensorimotor associations, mental rotation, linguistic processing.

In the present study we tested the hypothesis that there is a functional topography of M1 activation in studies where it has been implicated in higher cognitive tasks belonging to six different categories, namely: motor imagery, working memory, mental rotation, social/emotion/empathy, semantic and lexical decision, auditory processing. The six categories activated different sub-sectors of M1, either bilaterally or lateralized to one hemisphere. This finding strengthens the idea that there is a functional topography of M1 activation in studies where it has been found activated in higher cognitive tasks. The involvement of parts of M1 in cognitive processing has been confirmed by brain lesion studies on patients and by those using virtual lesions caused by TMS demonstrating that a lesion to M1 can lead to a deficit in motor imagery of hand rotational movements, (e.g., Ganis et al., 2000; Tomasino et al., 2011).

We acknowledge that areas 4a and 4p highly vary across participants and as a consequence the degree of smoothing is a relevant issue. For example, Sharma et al. (2008) used a small smoothing kernel (6 mm) while addressing areas 4a and 4p. This limitation of the study also to having included data from both fMRI and PET suggests that further studies are needed. However, the GingerALE method have been previously used to address functional topografy in areas such as the insula (Kurth et al., 2010) and the cerebellum (Stoodley and Schmahmann, 2009) with the same limitation.

### A view on M1 activation during non-motor and cognitive tasks

The reason for M1 activation in cognitive processing could be related to mental simulation processing triggered by the task either implicitly or explicitly, or to motor attention mechanisms. Motor simulation entails the rehearsal of a motor task and can occur explicitly or can be triggered implicitly (Jeannerod and Frak, 1999). An example of implicit triggering motor imagery is whensubjects implicitly imagine an action, with no instruction to do so, while performing for instance mental rotation of body parts (Zacks et al., 1999; Kosslyn et al., 2001), handedness recognition of a visually presented hand (Parsons and Fox, 1998), judgment as to whether an action would be easy, difficult or impossible (Johnson et al., 2002), or recognition actions performed by others (Jeannerod and Frak, 1999). M1 may contribute to the different cognitive processes through its role in anticipatory/implicit mental simulation or motor attention processing during somatotopic, dynamic remapping, a process in which individuals mentally track and continuously update the transformation of the body part motor image. Accordingly, the concept of internal models is used to predict sensory events, relations and states of other agents in the environment (Wolpert et al., 2003; Grush, 2004; Ito, 2008; Imamizu and Kawato, 2009). Alternatively, M1 activation is modulated by a top-down influence of cognitive strategies used to carry out the tasks (Tomasino and Rumiati, 2013). Evidence for this explanation could be found in the results from the six categories as described below.

### M1 activation within the six cognitive domains

For instance, we found that M1 is involved in language processing. The M1 activation during action-related word processing has been related to processing demands emphasizing the motor features of the verbs since no motor activation was found when the task instructions stressed visual rather than motor information (Kable et al., 2002). Similarly, it has been proposed that M1 activation could result from the subjects' strategy to mentally simulate the movements described by the verbs while processing language or from the context in which action words are presented (Tomasino and Rumiati, 2013).

Our meta-analysis also confirmed the activation of part of M1, namely Area 4a, during social/empathic/emotion processing. In line with the above assumptions, the M1 activation found during social/empathic/emotional processing could result from a co-activation of the motor circuitry because action schemes expressing the perceived emotion trigger an implicit simulation. Authors such as (Lamm et al., 2007) found a direct correlation between sensorimotor activation and empathy for pain. In their study, participants were presented pictures of painful and non-painful needle injections and were asked to rate their own perception of pain. One possibility could be that participants imagined themselves performing the same proposed painful task and their brain imagined an escape movement from the painful stimulation (e.g., by imagining turning the head away or performing a step backward) which involved a conspicuous set of body movements. Accordingly, it has been proposed that the precentral gyrus and M1 should be included in the empathic circuit (Guo et al., 2012). M1 activation has also been found during silent reading of emotional words (Papeo et al., 2012).

The view that M1 activation could be dependent on the strategy used by subjects while processing the task also relies on evidence of M1 activation in the mental spatial transformation category. Deciding whether objects, hands and alphanumeric characters were the same or mirrored images triggered M1 activation, possibly because subjects could mentally move the images in the same way as they would physically do by using their hands (Kosslyn et al., 2001). For example, (Kosslyn et al., 2001) showed that the left M1 was selectively activated *only* when subjects were explicitly asked to imagine grasping and turning a 3D object with their own hand (i.e., motor strategy), but not when they just imagined the object rotating in the visual field (i.e., visual strategy).

Furthermore, in the working memory domain, the activation found in Area 4a during recall of sensory material or of finger movements sequences after a time delay (Kaas et al., 2007), which was confirmed by our meta-analysis, has been related to holding a sensory item or movements on-line. It has been shown that remembering after a time delay whether a dot still appeared in the same position as before activated M1 possibly due to the transformation of visual coordinates into motor coordinates (for example, performing a saccade or a grasp) and to the retention of these coordinates during the time delay (Postle, 2006).

In addition, in the auditory processing domain, the activation found in Area 4a during passive listening of speech (Wilson and Iacoboni, 2006), which was confirmed by our meta-analysis, has been related to the generation of an internal model of speech sound under consideration (Hickok et al., 2011).

Lastly, and complementary to the above view, our meta-analysis confirmed the role of Areas 4a and 4p in motor imagery. Motor imagery is defined as the mental rehearsal of a motor act that occurs in the absence of overt motor input and the essential component of motor imagery is that the subject imagines him/herself executing the action from a first-person perspective without a real movement execution (Ehrsson et al., 2003). M1 activation has been related to first person perspective-taking during simulation of body movements or to the distinction between the self and the others (Ruby and Decety, 2001) or to the level of vividness of mental imagery (Lorey et al., 2011). It has been proposed that motor imagery could be the body-based simulator that relies on the sensorimotor system as its essential substrate (Lorey et al., 2011) in a somatotopic manner, (e.g., Ehrsson et al., 2003). Activation in M1 has been confirmed also in a multivariate fMRI analysis in which an independent component for motor imagery in area 4 was found. Unfortunately, no further distinction between area 4p or 4a was done since it was not appropriate considering these areas as separate due to the degree of smoothing required by the analysis authors performed (Sharma and Baron, 2013).

To sum up, area 4a or area 4p (or both) are found consistently activated for the analyzed categories. We acknowledge that a meta-analysis of fMRI and PET data does not account for the limitation of these techniques to discriminate the temporal sequence in which activations in area 4a and 4p may occur, leaving open the possibility that both areas may be activated but not at the same time. Nevertheless, an ALE meta-analysis grants (statistically) for the consistency of activation across a large body of data. However, further studies employing different techniques such as (MEG/EEG localization studies or TMS) might be able to disentangle the role of each M1 sector.

### Conjunction analysis

The conjunction analysis investigated whether a sector of M1 was commonly activated by all the six categories. Our analysis revealed that an area located in the left Area 4a at the MNI coordinates *x, y, z* = −35, −25, +62 was conjointly activated by 4 out of 6 categories namely motor imagery and working memory, social/emotion/empathy, and language. The coordinates are also very close to those reported in a previous study in which a hand movement localizer task was used (mean MNI coordinates: −38, −26, 60, Papeo et al., 2012) and to peak coordinates: −38, −30, 66 (Area 4a of hand M1, Papeo et al., 2012) reported for the greater activity they showed during a motor vs. a visuospatial imagery task and to those (−30, −24, 62, Papeo et al., 2012) reported for the greater activity they showed during a reading action or state verb reading in the motor context (after performing a motor strategy based mental rotation) vs. reading in the non-motor context (after performing a visuospatial strategy-based mental rotation). All meta-analyses showed activation within area 4a except for the mental imagery task which also showed activation in area 4p, and the area commonly activated by all categories was Area 4a.

In a recent review on the agranular structure of M1 it is restated that the functions of M1 are due to the lack of a major pathway ascending through area 4 to area 6, to the absence of layer 4 and a thinner layer 3. In particular, the connections are possible from area 6 to area 4. This connection is responsible for a modulatory effect exerted by area 6 on area 4. In addition, the connections from the somatosensory cortex are important for feeding M1 with a kinesthetic information in order to select the appropriate effectors (Shipp, 2005). It has been shown that M1 is divided in two areas 4a and 4p on the basis of anatomy, neurochemistry, and function (Geyer et al., 1996). From a neurochemical point of view, areas 4p and 4a significantly differ. Geyer et al. (1996) showed that laminar density of neurons in area 4p and 4a significantly differ as well as receptors is concerned. In addition authors (Geyer et al., 1996) found that area 4a has larger pyramidal cells in layer III and more densely packed as compared to area 4p. The two sectors of M1 present also different connectivity patterns. It has been suggested that while area 4p is more similar to the primary sensory cortex, area 4a more similar to the premotor cortex. Evidence for this view came from fMRI studies. In one study, a roughness discrimination task between two cylinders with different roughness performed by using the right thumb and index activated area 4p more than self-generated movements without object interaction (Geyer et al., 1996; Geyer, 2004). Geyer (2004) argued that a voluntary motor act that is modulated by somatosensory feedback (roughness discrimination task) stronger activates area 4p. In another study, activation in the same area 4p has been found to be modulated by attention to action, while activation in area 4a was not (Binkofski et al., 2002). Similarly, other authors (Johansen-Berg and Matthews, 2002) showed that a concurring distraction task i.e., counting backwards, performed while subjects attended to movements, reduced activation in area 4p. The functional dissociation we described in the present study might well-complement the functional differences reported above. Indeed, we found that several cognitive tasks, likely triggering an implicit motor imagery, activated the other sector of M1, that is area 4a. This result is consistent with an fMRI study addressing motor imagery of different body parts (Ehrsson et al., 2003) showing that imagery of hands and feet movements (flexion-extension) preferentially activated area 4a. In another fMRI study however, motor imagery of finger movements activated both areas 4a and 4p, and the latter was more activated than area 4p similar to the levels of activation found in real execution of movements (Sharma et al., 2008). Our results complements this pattern adding further evidence that area 4a could be involved in cognitive tasks. It has been shown that while the posterior Area 4p is connected with the primary sensory cortex, the anterior area 4a is connected with the premotor cortex (Stepniewska et al., 1993). This connectivity patterns is in line with the view that area 6 exerts his influence on area 4a activation during motor imagery (Passingham, 1997).

One view is that a simply stronger connection between premotor and motor cortex exists due to just handedness. In our dataset only studies involving right handed participants were included, thus an additional meta-analysis on studies that used or left handed subjects (and left hand responses in right handed subjects) is missing and could be the topic of a further study.

There is evidence, for instance, in the motor imagery domain, that during the mental simulation of hand actions a left lateralized activation in areas involved in motor planning and execution was found in right handed participants, whereas a right lateralization in the same areas was found in left-handed participants (Willems et al., 2010).

Another view is that the left lateralization is due to hemispheric specialization, and in this view, results are likely to reflect the LH dominance for action and goal-directed motor behavior (and apraxia).

## Conclusion

Results showed that there is a functional topography of M1 activation in studies where it has been found activated in higher cognitive tasks. For all the categories M1 activation could be related to mental simulation as strategy used by subjects (implicitly or explicitly) to solve the task. A commonly activated sector shared by all the categories found in Area 4a could be considered as a hub for the cognitive role of the motor cortex area. Few studies support an active role of Area 4a in cognitive processing. However, the low number of studies reporting M1 activations during cognitive tasks is also imputable to methodological reasons. For instance, if cytoarchitectonically defined maximum probability maps (MPMs) in the standard anatomical space are not used, it is not possible to define the border between M1 and the posterior part of Area 6. Further studies are necessary to confirm these activations. Thus, our meta-analysis supports the notion that area 4a function can go beyond simple motor output.

## Author contributions

BT contributed to the conception, design, preparation, analysis and interpretation of the data. MG contributed to the preparation, analysis, and interpretation of the data.

## Funding

BT was supported by grants from the IRCCS “E. Medea” (Ricerca Corrente).

### Conflict of interest statement

The authors declare that the research was conducted in the absence of any commercial or financial relationships that could be construed as a potential conflict of interest.

## Footnotes

^1^We considered this category as “cognitive” since in the included studies participants were asked to perform a task after or during listening, e.g., discrimination task, judgments, or imagery (see Jardri et al., 2007).
