# Supplementary material for: The Cognitive Side of M1
Source: Front Hum Neurosci. 2016 Jun 17;10:298. doi: 10.3389/fnhum.2016.00298 (PMC4911410; doi:10.3389/fnhum.2016.00298)
Supplement: Supplementary file 1 [file Table1.DOC]

**Supplemental Table S1**

**Details about the key presses**

| **Category** | **N studies** | **N studies with button presses** | **R** | **L** | **both** | **N.R.** | **M1 activation lateralized to** |
| --- | --- | --- | --- | --- | --- | --- | --- |
| ***Social/Emotion /Empathy*** | 19 | 12 | 4 (33.33%) |  | 2 (16.66%) | 6 (50%) | **L** |
| ***Action word/verb processing*** | 32 | 15 | 8 (53.33%) | 3 (20%) | 1 (6.66%) | 3  (20%) | both |
| ***Mental Rotation*** | 10 | 10 | 1 (10%) |  | 6 (60%) | 3 (30%) | **L** |
| ***Working Memory*** | 29 | 23 | 15 (65.21%) |  | 5 (21.73%) | 3 (13.04%) | both |
| ***Motor Imagery*** | 22 | 4 | 1 (25%) | 1 (25%) | 2 (50%) | - | both |
| ***Auditory*** | 14 | 4 | 2 (50%) | - | 2 (50%) | - | **L** |

**Supplementary Figure 1**

**The anatomical mask created by using Anatomy toolbox on the left and right Area 4a and 4p**

**
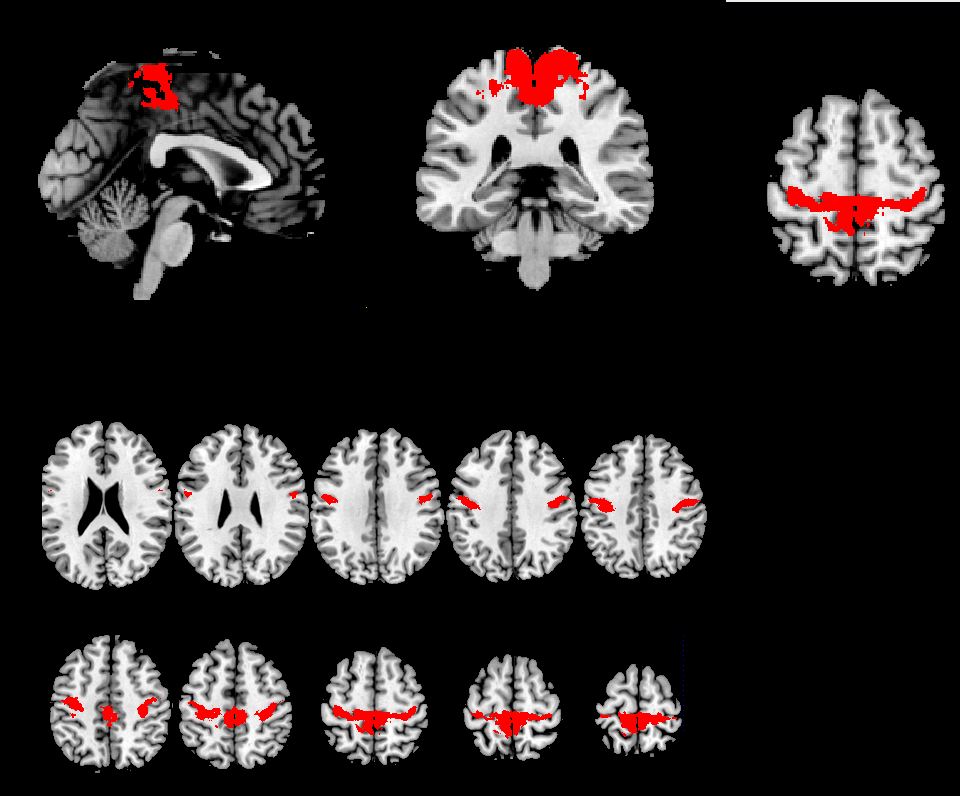
**

**Supplementary Figure 2**

Network of activations underlying the different categories analysed. Relative increases in neural activity associated with the tasks are displayed on a rendered template brain. Activations are significant at p <.05 corrected for multiple comparisons using the False Discovery Rate (FDR).

**
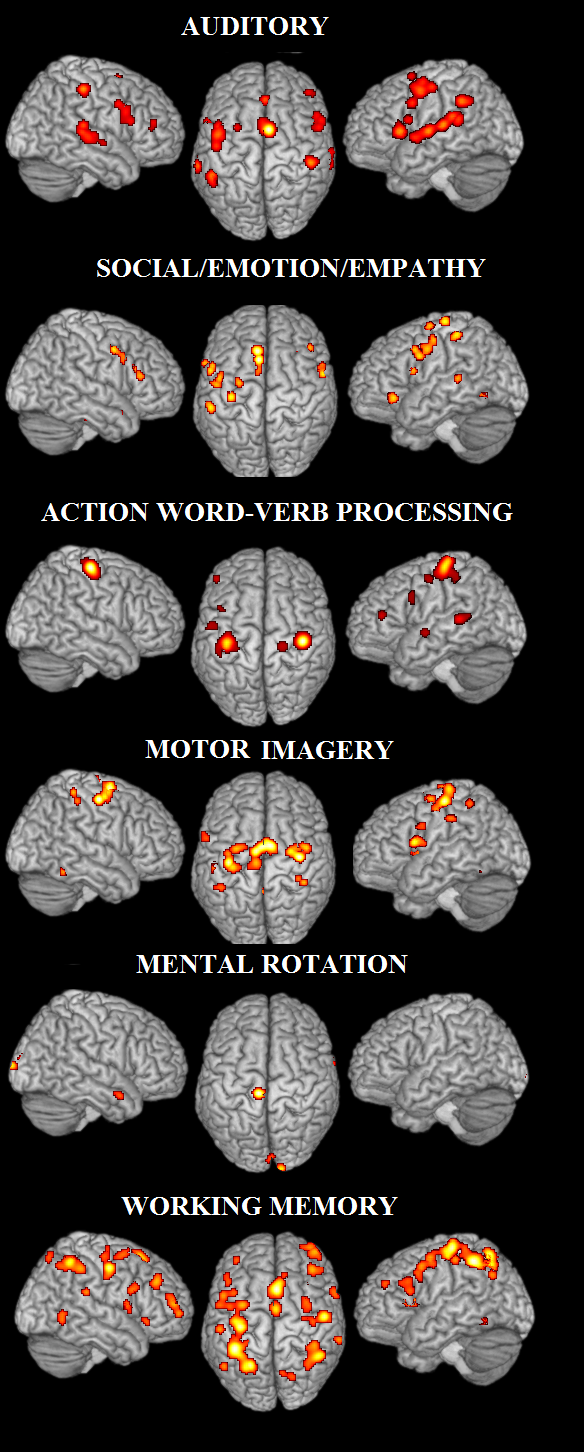
**

**Supplementary Figure 3**

Sagittal slices of the activations in M1 related to the different categories.


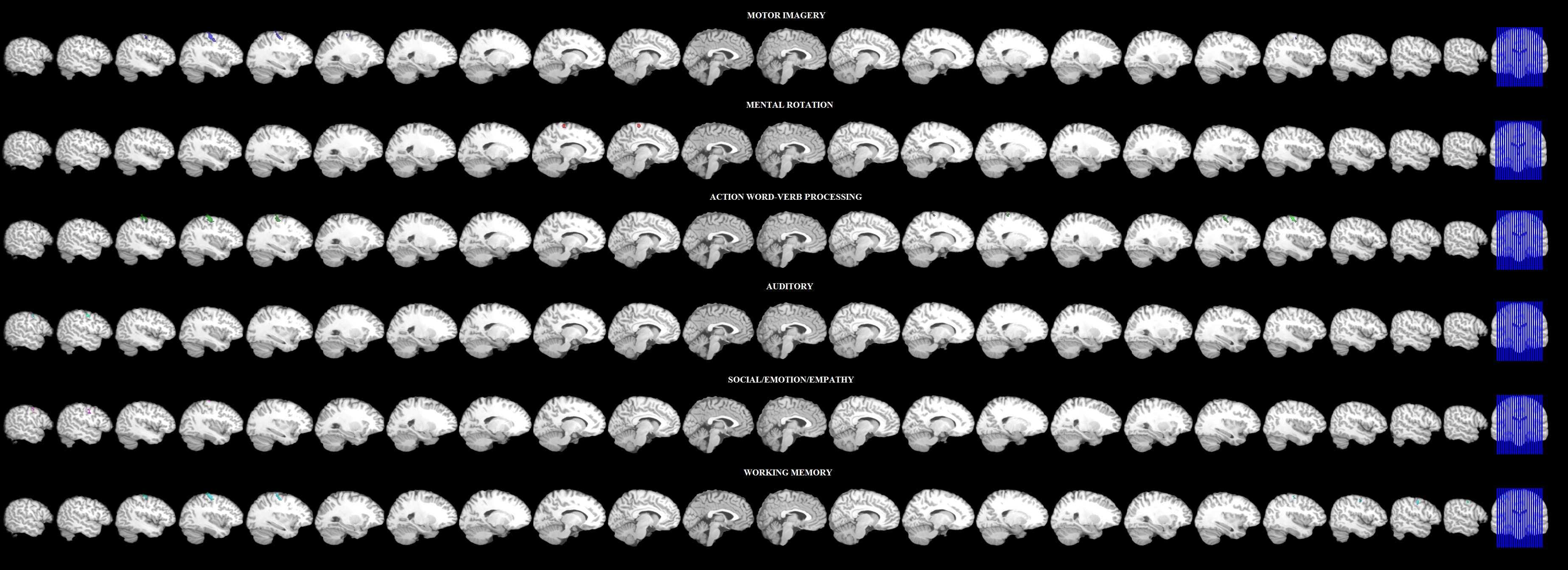


Supplemental Table S2

| ***ACTION WORD/VERB PROCESSING*** | | | | | | | |
| --- | --- | --- | --- | --- | --- | --- | --- |
| **Cluster** |  | **Area** | **MNI** | | | **Cluster size (voxels)** | **Extrema value** |
|  |  |  | **X** | **Y** | **Z** |  |  |
| 1 | L | Postcentral Gyrus, probability for Area 4p 20%, probability for Area 3b 30%, probabilty for Area 4a 40% | -38 | -24 | 58 | 607 | 0.069 |
| 2 | R | Precentral Gyrus, probability for Area 4a 50%, probability for Area 6 30% | 38 | -22 | 58 | 454 | 0.092 |
| 3 | L | Middle Temporal Gyrus | -56 | -38 | 2 | 112 | 0.035 |
| 4 | L | Postcentral Gyrus, probability for Area 6 50%,  probability for Area 4a 50% | -54 | -8 | 42 | 54 | 0.023 |
| 5 | R | Precentral Gyrus, probability for Area 6 30%, probability for Area 4p 30%, probability for Area 4a 20% | 18 | -28 | 64 | 53 | 0.026 |
| 6 | L | Inferior Frontal Gyrus, probability for Area 44 50% | -44 | 10 | 26 | 33 | 0.021 |
| 7 | L | Inferior Frontal Gyrus, probability for Area 45 20% | -48 | 40 | 6 | 32 | 0.028 |
| 8 | L | Superior Frontal Gyrus | -58 | -4 | -14 | 29 | 0.024 |

Supplemental Table S3

| ***SOCIAL / EMOTION / EMPATHY*** | | | | | | | |
| --- | --- | --- | --- | --- | --- | --- | --- |
| **Cluster** |  | **Area** | **MNI** | | | **Cluster size (voxels)** | **Extrema value** |
|  |  |  | **X** | **Y** | **Z** |  |  |
| 1 | L | SMA. probability for Area 6 20% | -8 | 14 | 48 | 253 | 0.024 |
| 2 | L | Precentral Gyrus. probability for Area 4a 10% | -46 | -4 | 34 | 121 | 0.016 |
| 3 | R | Amygdala. probability for Amyg (LB) 40%. probability for Hipp (EC) 20%. probability for Amyg (SF) 20% | 24 | 0 | -24 | 112 | 0.022 |
| 4 | R | Precentral Gyrus. probability for Area 6 70% | 56 | 0 | 40 | 103 | 0.018 |
| 5 | L | Precentral Gyrus. assigned to Area 44 30%. probability for Area 6 20% | -56 | 4 | 24 | 77 | 0.017 |
| 6 | L | Thalamus | -14 | -14 | 10 | 74 | 0.018 |
| 7 | L | Inferior Parietal Lobule. assigned to IPC (PF) 80%. probability for IPC (PFt) 50% | -56 | -32 | 46 | 73 | 0.018 |
| 8 | L | Amygdala. probabiility for Amyg (LB) 80%. probability for Amyg (SF) 70% | -20 | -6 | -16 | 61 | 0.019 |
| 9 | L | Precentral Gyrus. probability for Area 6 80%. probability for area 1 20%. probability for Area 4a 9,2% | -36 | -22 | 64 | 60 | 0.019 |
| 10 | L | Frontal Gyrus (p. Orbitalis). probability for Area 45 10% | -44 | 30 | -16 | 53 | 0.017 |
| 11 | R | Not found in any probability map | 32 | 18 | 22 | 49 | 0.017 |
| 12 | R | Parahippocampal Gyrus. probability for Hipp (CA) 90%. probability for Hipp (SUB) 80% | 30 | -24 | -16 | 46 | 0.015 |
| 13 | R | Thalamus. probability for Hipp (FD)40%. probability for Hipp (CA) 30% | 20 | -30 | 2 | 46 | 0.016 |
| 14 | R | Frontal Gyrus (p. Triangularis). probability for Area 45 10% | 44 | 26 | 14 | 39 | 0.015 |
| 15 | R | Cerebellum | 26 | -24 | -28 | 39 | 0,015 |
| 16 | L | Fusiform Gyrus | -38 | -62 | -12 | 35 | 0.016 |
| 17 | L | Inferior Temporal Gyrus | -38 | -42 | -16 | 35 | 0.015 |
| 18 | L | Superior Frontal Gyrus. probability for Area 6 40% | -28 | -8 | 62 | 34 | 0.016 |
| 20 | R | Insula Lobe | 30 | 26 | 4 | 31 | 0.015 |
| 21 | L | Middle Temporal Gyrus. probability for IPC (PF) 10% | -58 | -34 | 2 | 27 | 0.015 |
| 22 | L | Inferior frontal gyrus | -62 | 12 | 8 | 25 | 0.015 |

Supplemental Table S4

| ***MENTAL ROTATION*** | | | | | | | |
| --- | --- | --- | --- | --- | --- | --- | --- |
| **Cluster** |  | **Area** | **MNI** | | | **Cluster size (voxels)** | **Extrema value** |
|  |  |  | **X** | **Y** | **Z** |  |  |
| 1 | L | Paracentral Lobule. probability for Area 4a 50%; probability for Area 6 30%; | -10 | -28 | 64 | 120 | 0.019 |
| 2 | R | probability for Area 17, 80%  probability for Area 18 20% | 12 | -104 | 4 | 47 | 0.014 |
| 3 | R | Middle temporal gyrus | 62 | 4 | -26 | 37 | 0.010 |
| 4 | L | Cuneus, probability for Area 18 10% | 4 | -92 | 14 | 28 | 0.009 |

Supplemental Table S5

| ***WORKING MEMORY*** | | | | | | | |
| --- | --- | --- | --- | --- | --- | --- | --- |
| **Cluster** |  | **Area** | **MNI** | | | **Cluster size (voxels)** | **Extrema value** |
|  |  |  | **X** | **Y** | **Z** |  |  |
| 1 | L | Inferior Parietal Lobule. assigned to hlP1 30% | -40 | -46 | 46 | 1856 | 0.031 |
| 2 | R | SMA. probability for Area 6 20% | 2 | 14 | 50 | 498 | 0.031 |
| 3 | R | Inferior Parietal Lobule. probability for IMP (Pga) 40% | 44 | -52 | 50 | 460 | 0.025 |
| 4 | R | Precentral Gyrus. probability for Area 4a 50% | 50 | -14 | 44 | 276 | 0.028 |
| 5 | R | Middle Frontal Gyrus | 40 | 52 | 8 | 267 | 0.020 |
| 6 | R | Right Middle Frontal Gyrus | 38 | 34 | 30 | 159 | 0.020 |
| 7 |  | SMA. probability for Area 6 80% | 2 | 14 | 50 | 147 | 0.023 |
| 8 | L | Insula Lobe | -30 | 18 | 6 | 133 | 0.025 |
| 9 | L | Frontal Gyrus (p. Triangularis). assigned to Area 44 30% | -46 | 20 | 14 | 108 | 0.017 |
| 10 | R | Middle Frontal Gyrus | 32 | 0 | 58 | 94 | 0.017 |
| 11 | R | Inferior Temporal Gyrus | 54 | -60 | -6 | 88 | 0.018 |
| 12 | R | Superior Parietal Lobule. assigned to SPL (7P) 70% | 18 | -74 | 54 | 81 | 0.016 |
| 13 | R | Insula Lobe | 40 | 4 | 8 | 80 | 0.018 |
| 14 | R | Inferior Frontal Gyrus Probability for Area 44, 40% | 60 | 10 | 22 | 73 | 0.022 |
| 15 | L | Inferior Parietal Lobule. assigned to Area 3 60% | -54 | -26 | 44 | 47 | 0.019 |
| 16 | R | Superior Temporal Gyrus. assigned to IPC (PF). probability 90% | 64 | -36 | 20 | 43 | 0.019 |
| 17 | L | Angular Gyrus. probability for IPM (Pga) 40% | -50 | -62 | 30 | 37 | 0.018 |
| 18 | L | Fusiform Gyrus | -42 | -54 | -16 | 34 | 0.018 |
| 19 | L | middle frontal gyrus | -38 | 44 | 22 | 30 | 0.016 |
| 20 | R | middle cingulated cortex | 10 | 28 | 36 | 25 | 0.015 |

**Supplemental Table S6**

| ***MOTOR IMAGERY*** | | | | | | | |
| --- | --- | --- | --- | --- | --- | --- | --- |
| **Cluster** |  | **Area** | **MNI** | | | **Cluster size (voxels)** | **Extrema value** |
|  |  |  | **X** | **Y** | **Z** |  |  |
| 1 | R | SMA. probability for Area 6 90% | 6 | -4 | 62 | 661 | 0.027 |
|  | L | SMA. probability for Area 6 80% | -2 | -4 | 64 |  | 0.027 |
|  | L | SMA. probability for Area 6 60% | -8 | -8 | 62 |  | 0.026 |
|  | L | Paracentral Lobule. probability for area 4a 30% | -8 | -20 | 70 |  | 0.018 |
|  | R | middle cingulte cortex. probability for Area 6 70% | 2 | -8 | 50 |  | 0.011 |
| 2 | R | Precentral Gyrus. probability for Area 6 60% | 34 | -14 | 52 | 407 | 0.027 |
|  | R | Probability for Area 6 20% | 26 | -10 | 54 |  | 0.021 |
|  | R | Middle Frontal Gyrus. proability for Area 6 40% | 42 | -6 | 58 |  | 0.019 |
|  | R | Middle Frontal Gyrus. probability for Area 6 20% | 28 | -8 | 60 |  | 0.018 |
| 3 | L | Precentral Gyrus. probability for Area 4p 40%. probability for Area 4a 40% | -36 | -20 | 52 | 467 | 0.027 |
|  | L | Precentral Gyrus. probability for Area 4a 20% | -32 | -24 | 66 |  | 0.020 |
|  | L | Precentral Gyrus. probability for Area 4a 30%. probability for Area 4p 20% | -36 | -12 | 44 |  | 0.016 |
| 4 | L | Cerebellum. probability for Lobules I-IV (Hem) 4% | -6 | -50 | -18 | 151 | 0.022 |
|  | L | Cerebellum. probability for Lobules I-IV (Hem) 46% | -4 | -50 | -4 |  | 0.017 |
| 5 | L | Left Inferior Frontal Gyrus (p. Opercularis). probability for Area 44 20% | -40 | 10 | 10 | 137 | 0.023 |
|  | R | Not found any probability map | 24 | -10 | 2 |  | 0.017 |
|  | R | Putamen | 28 | -10 | 10 |  | 0.016 |
|  | R | Putamen | 28 | -2 | -8 |  | 0.014 |
| 6 | R | Not found any probability map | 20 | 4 | 10 | 232 | 0.022 |
| 7 | R | Cerebellar Vermis. probability for Lobule VI (Hem) 73% | 6 | -68 | -18 | 99 | 0.020 |
| 8 | L | Putamen | -26 | -6 | 12 | 98 | 0.022 |
|  | L | Thalamus | -18 | -10 | 6 |  | 0.014 |
|  | L | Precentral Gyrus. probability for Area 4a 30%. probability for Area 4p 20% | -28 | -6 | 56 |  | 0.019 |
| 9 | L | Putmen | -22 | 4 | 2 | 81 | 0.016 |
| 10 | R | Postcentral Gyrus. assigned to Area 2 90% | 40 | -40 | 58 | 66 | 0.018 |
|  | R | Postcentral Gyrus. assigned to Area 2 90% | 34 | -36 | 50 |  | 0.015 |
| 11 | L | Lingual Gyrus. probability for hOC4v (v4) 30% | -14 | -64 | -8 | 48 | 0.016 |
| 12 | L | inferior Parietal Lobule. probability for IPC (PF) 30% | -44 | -46 | 48 | 41 | 0.014 |
| 13 | L | Fusiform Gyrus. probability for Lobule VI (Hem)1% | -34 | -56 | -18 | 38 | 0.015 |
| 14 | L | Supramarginal Gyrus. assigned to IPC (PFt) 50% | -50 | -26 | 32 | 35 | 0.015 |
| 15 | L | Precentral Gyrus. assigned to Area 44 30% | -58 | 4 | 22 | 34 | 0.014 |
| 16 | R | Fusiform Gyrus | 38 | -50 | -22 | 29 | 0.017 |
| 17 | R | Not found any probability map | 20 | -26 | 18 | 29 | 0.017 |

**Supplemental Table S7**

| ***AUDITORY*** | | | | | | | |
| --- | --- | --- | --- | --- | --- | --- | --- |
| **Cluster** |  | **Area** | **MNI** | | | **Cluster size (voxels)** | **Extrema value** |
|  |  |  | **X** | **Y** | **Z** |  |  |
| 1 | R | SMA. probability fot Area 6 60% | 4 | 2 | 54 | 344 | 0.037 |
| 2 | L | Precentral Gyrus. probability for Area 6 20% | -46 | -2 | 40 | 256 | 0.020 |
|  | L | Postcentral Gyrus. probability for Area 3b 40%. probability for Area 1 30%. probability for Area 4a 30% | -48 | -14 | 44 |  | 0.018 |
|  | L | Precentral Gyrus. probability for Area 6 70%. probability for Area 4a 10% | -44 | -4 | 50 |  | 0.017 |
|  | L | Precentral Gyrus. probability for Area 44 10% | -46 | 6 | 44 |  | 0.012 |
| 3 | L | Superior Temporal Gyrus. probability for TE 1.2 10%. probability for TE 1.0 10% | -56 | -10 | -2 | 406 | 0.024 |
|  | L | Ssuperior Temporal Gyrus. probability for OP 1 10%. probability for TE 1.0 10% | -60 | -24 | 2 |  | 0.024 |
|  | L | superior Temporal Gyrus. probability for IPC (PF) 10%. probability for IPC (Pfop) 10% | -66 | -32 | 10 |  | 0.020 |
|  | L | Superior Temporal Gyrus | -54 | 4 | -8 |  | 0.015 |
| 4 | L | Left Insula Lobe. probability for Area 45 10%. probability for Area 44 10% | -42 | 18 | 0 | 166 | 0.024 |
| 5 | R | Inferior Frontal Gyrus. probability for Area 44 60%. probability for Area 45 10% | 56 | 12 | 24 | 154 | 0.016 |
|  | R | Precentral Gyrus. probability for Area 44 10%. probability for Area 6 10% | 54 | 6 | 32 |  | 0.015 |
|  | R | Inferior Frontal Gyrus. probability for Area 44 50%. probability for Area 45 40% | 52 | 18 | 16 |  | 0.014 |
| 6 | R | Superior Temporal Gyrus. probability for IPC (PF) 20% | 64 | -32 | 6 | 131 | 0.016 |
|  | R | Superior Temporal Gyrus | 56 | -22 | -4 |  | 0.013 |
| 7 | R | Postcentral Gyrus. probability for IPC (PFt) 40%. probability for Area 1 20% | 46 | -30 | 48 | 125 | 0.024 |
| 8 | L | SupraMarginal Gyrus. probability for IPC (PFcm) 70%. probability for IPC (PF) 30%. probability for IPC (PFm) 30% | -52 | -42 | 28 | 102 | 0.021 |
| 9 | L | Middle Temporal Gyrus | -52 | -40 | 10 | 91 | 0.019 |
| 10 | R | Superior Temporal Gyrus. probability for TE 1.1 30%. probability for IPC (PFcm) 10% | 44 | -30 | 12 | 41 | 0.014 |
| 11 | L | Left Inferior Frontal Gyrus. probability for Are 44 40%. probability for Area 45 10%. probability for Area 3b 10% | -58 | 10 | 8 | 30 | 0.015 |
| 12 | L | Caudate Nucleus | -16 | -6 | 20 | 29 | 0.015 |
| 13 | L | Heschls Gyrus. probability for TE 1.1 90%. probability for TE 1.0 20% | -40 | -28 | 10 | 25 | 0.014 |
